# Comparative neuroanatomy suggests repeated reduction of neuroarchitectural complexity in Annelida

**DOI:** 10.1186/1742-9994-7-13

**Published:** 2010-05-04

**Authors:** Carsten M Heuer, Carsten HG Müller, Christiane Todt, Rudi Loesel

**Affiliations:** 1 Institute for Biology II, RWTH Aachen, Department of Developmental Biology and Morphology of Animals, D-52056 Aachen, Germany; 2 Ernst -Moritz-Arndt-Universität Greifswald, Zoologisches Institut, Cytologie und Evolutionsbiologie, Johann-Sebastian-Bach-Straße 11/12, D-17487 Greifswald, Germany; 3 University of Bergen, Department of Biolog y, Thormøhlensgate 53b, 5008 Bergen, Norway

## Abstract

**Background:**

Paired mushroom bodies, an unpaired central complex, and bilaterally arranged clusters of olfactory glomeruli are among the most distinctive components of arthropod neuroarchitecture. Mushroom body neuropils, unpaired midline neuropils, and olfactory glomeruli also occur in the brains of some polychaete annelids, showing varying degrees of morphological similarity to their arthropod counterparts. Attempts to elucidate the evolutionary origin of these neuropils and to deduce an ancestral ground pattern of annelid cerebral complexity are impeded by the incomplete knowledge of annelid phylogeny and by a lack of comparative neuroanatomical data for this group. The present account aims to provide new morphological data for a broad range of annelid taxa in order to trace the occurrence and variability of higher brain centers in segmented worms.

**Results:**

Immunohistochemically stained preparations provide comparative neuroanatomical data for representatives from 22 annelid species. The most prominent neuropil structures to be encountered in the annelid brain are the paired mushroom bodies that occur in a number of polychaete taxa. Mushroom bodies can in some cases be demonstrated to be closely associated with clusters of spheroid neuropils reminiscent of arthropod olfactory glomeruli. Less distinctive subcompartments of the annelid brain are unpaired midline neuropils that bear a remote resemblance to similar components in the arthropod brain. The occurrence of higher brain centers such as mushroom bodies, olfactory glomeruli, and unpaired midline neuropils seems to be restricted to errant polychaetes.

**Conclusions:**

The implications of an assumed homology between annelid and arthropod mushroom bodies are discussed in light of the 'new animal phylogeny'. It is concluded that the apparent homology of mushroom bodies in distantly related groups has to be interpreted as a plesiomorphy, pointing towards a considerably complex neuroarchitecture inherited from the last common ancestor, Urbilateria. Within the annelid radiation, the lack of mushroom bodies in certain groups is explained by widespread secondary reductions owing to selective pressures unfavorable for the differentiation of elaborate brains. Evolutionary pathways of mushroom body neuropils in errant polychaetes remain enigmatic.

## Background

Annelida is an ancient phylum that comprises over 16,500 described species. Its members inhabit nearly all biotopes in marine environments, and occupy fresh water and moist terrestrial habitats [1]. Traditionally, the segmented worms are thought to fall into two major groups: Polychaeta (bristleworms) and Clitellata (worms with a specialized reproductive structure, the clitellum). Among them, Polychaeta represents the larger and more diverse taxon, a fact that can be attributed to the high evolutionary plasticity of the polychaete body plan. In different groups, head appendages (prostomial palps and tentacles, peristomial cirri) and body appendages (parapodia) have been modified in numerous ways to suit a wide range of lifestyles and feeding strategies, including active predation, scavenging, deposit and suspension feeding. Clitellata, in contrast, lack elaborate head and body appendages and thus show less diverse body forms. While ectoparasitic members of the Hirudinea (leeches) have a specialized feeding apparatus that allow them to feed on the body fluids of their hosts, other clitellates can be characterized as predators, detritivors or direct deposit feeders.

Surprisingly, evolutionary relationships within this ecologically important metazoan phylum are still poorly understood [2-4]. Clitellatesare g enerally accepted to form a monophyletic clade, but phylogenetic relationships within the group are still a matter of debate [5]. Polychaete relationships represent one of the most intractable problems of phylogenetic research [6] and the history of polychaete systematics is accordingly long and convoluted [reviewed in [7]]. The most influential traditional concept divided the group into the orders Sedentaria and Errantia [8]. This division was later recognized as a rather arbitrary grouping [9], useful for practical purposes but not representing correct phylogenetic relationships. Due to the lack of conclusive evidence, many authors subsequently refrained from grouping the approximately 80 well-established polychaete families into higher-ranking taxa. Analyzing morphological character traits across a broad range of families, Rouse and Fauchald [10] provided one of the most comprehensive cladistic studies to date. They proposed the Polychaeta to form two major clades, Scolecida and Palpata, the latter comprising the Canalipalpata (containing the remainder of the Sedentaria) and the Aciculata (containing the remainder of the Errantia). However, not all of these groups are strongly supported [10,11] and their monophyly has been questioned by morphological [12,13] as well as molecular studies [2,4,14]. As yet, a conclusive phylogeny for the segmented worms is still lacking and annelid systematics remain "*one of the most vexing problems in invertebrate phylogenetics*" [[3], page 1462].

Searching for novel characters to advance the reconstruction of annelid relationships, several authors have looked into the architecture of the central nervous system. The metameric organization of the ventral nerve cord in developing stages of *Bonellia viridis*led Hessling [15] to propose the inclusion of echiurids into the Annelida. Similarly, segmental patterns observed during neurogenesis of *Phascolion strombus*[16] and *Phascolosoma agassizii *[17]indicate a close affinity of Sipuncula and Annelida. Detailed investigations into the neuroarchitecture and the internal scaffolding of the central nervous system across a wide range of polychaete species were provided by Orrhage and Müller [18]. Drawing on innervation patterns, these authors postulated homology hypotheses for the highly variable head appendages encountered in different polychaete species. In a similar approach, Zanol and Fauchald [19] presented in-depth neuroarchitectural descriptions of eunicid polychaetes to elucidate evolutionary transformations of the head appendages in this group.

**Figure 1:**
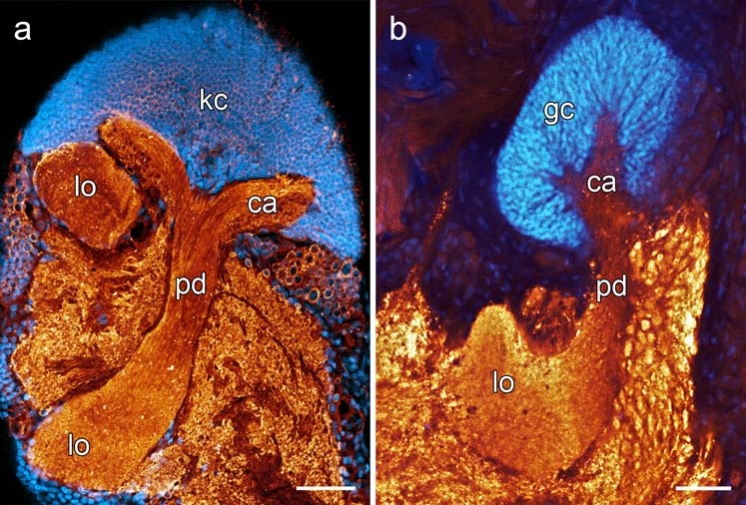
**Comparison of arthropod and annelid mushroom bodies**. (a) Horseradish peroxidase immunoreactivity and cell nuclei labeling in sagittal sections of the brain of the cockroach *Leucophaea maderae *reveal the basic neuropil organization. The mushroom body is capped by a dorsal aggregation of small-diameter Kenyon cells (**kc**) and comprises a calyx region (**ca**), a peduncle (**pd**), and an arrangement of median and vertical lobes (**lo**). (b) Synapsin immunoreactivity in cell nuclei labeled horizontal sections of the brain of the polychaete *Nereis diversicolor *shows a similar organization of the annelid mushroom body neuropil. **gc **globuli cells. Scale bars: 80 μm.

Little attention, however, has so far been devoted to the neuroanatomy of higher brain centers in annelids and their phylogenetic significance. The first accounts to describe such neuropil structures in a variety of annelid species were presented by Holmgren [20] and Hanström [21,22] who reported the occurrence of arthropod-like neuropils in the brain of certain polychaetes. Arguing along the lines of an assumed sistergroup relationship of annelids and arthropods (Articulata), these early authors interpreted the observed neuropil structures as homologues. However, in light of the recent rearrangement of metazoan lineages into Ecdysozoa [23] and Lophotrochozoa [24], and considering the occasionally low anatomical resolution presented in the early reports, a critical re-evaluation of this homology hypothesis seemed warranted. In a recent account, Heuer and Loesel [25] provided a detailed analyses of brain centers in the polychaete *Nereis diversicolor*, corroborating a high neuroanatomical similarity between the most prominent brain centers - the so-called mushroom bodies - in annelids and arthropods (Fig. 1). Indicators for homology are a common neuropil organization comprising distinctive substructures, a comparable neural connectivity and a presumably similar function of these brain centers [26]. Recent findings from the field of evolutionary developmental biology have shown the mushroom body anlagen in larvae of the nereid polychaete *Platynereis dumerilii *to express the same combination of genes that is characteristic for the developing mushroom bodies of *Drosophila melanogaster*, providing independent support for these anatomy-derived homology assessments [27].

**Figure 2:**
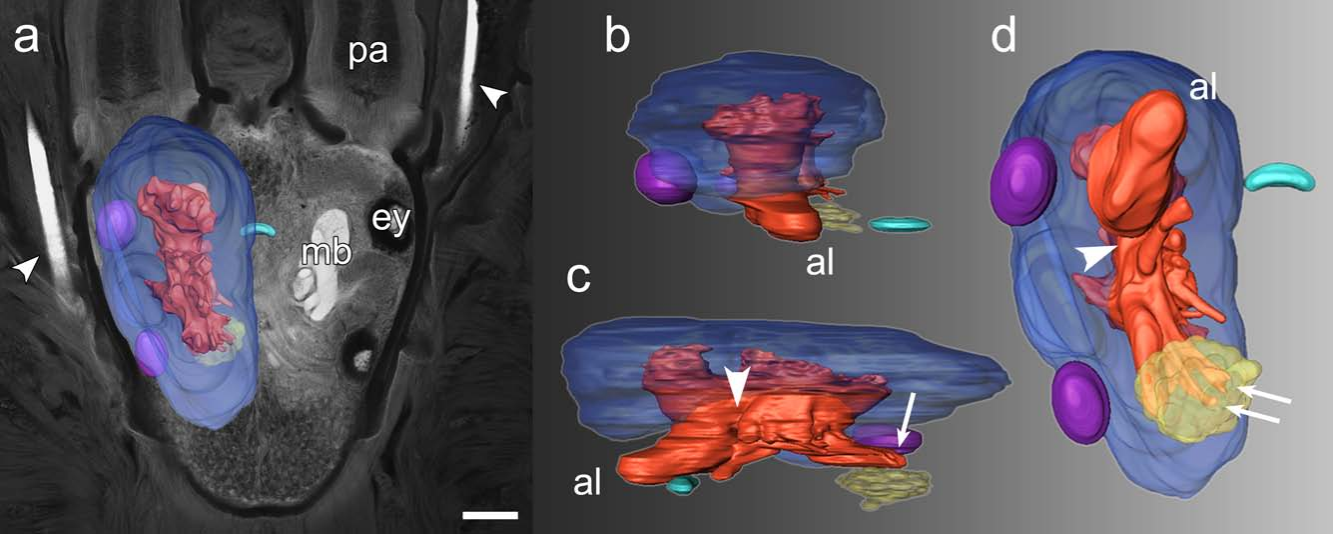
**Three-dimensional surface reconstruction of the mushroom body neuropil in the polychaete *Lepidonotus clava***. (a) Three-dimensional mushroom body model superimposed upon an autofluorescence image of a horizontal section through the head of the animal (anterior is towards the top of the picture). The contour of the prostomium provides a context to which the relative position and size of the clearly demarcated mushroom bodies (**mb**) can be related. In the reconstruction, the globuli cell cluster (**blue**) is colored transparently, allowing for the intricate arborizations of the mushroom body neuropile (**red**) to be seen. **pa **palp, **ey **eye (b, c, d) Anterior (b), median (c), and ventral (d) views of the surface reconstruction show a thick mass of globuli cell bodies surrounding most parts of the neuropil and forming indentations to accommodate the anterior and posterior eyes (**purple**). Where it is embedded in globuli cell somata, the neuropil forms protuberances. About the dorso-ventral midline (**arrowheads**), the neuropil proper splits into an anterior lobe (**al**) and a posterior part. While the anterior lobe shows a smooth, unbroken surface, the posterior part forms several extensions that establish contact with the central neuropil. Two of these extensions (**arrows**) connect to a cluster of glomeruli (**yellow**) that lies adjacent to the ventro-posterior part of the mushroom bodies. Also shown here is a crescent-shaped neuropil region (**green**) that lies between the mushroom bodies and spans the midline of the brain. Scale bar: 200 μm.

Continuative investigations into annelid mushroom body morphology (Fig. 2), utilizing3D reconstructions to compare the neuropil organization in *N. diversicolor *and the polynoid *Harmothoe areolata *[28], demonstrated morphological variability of these neuropils in different taxa and provided evidence for the correlation of anatomical difference with phylogenetic distance. Yet, in comparison to the amount of literature available on arthropod neuroanatomy, studies on the distribution and differentiation of higher brain centers in annelids are scarce, and attempts to trace the evolution of cerebral neuropils or to use neuroarchitectural characters for phylogenetic considerations are hampered by the lack of neuroanatomical data for a wide range of annelid representatives. The present study takes a step towards remedying this situation by providing comparative descriptions of higher brain centers in a broad variety of annelid taxa.

## Materials and methods

Polychaete specimens were collected during field trips to the Isle of Ibiza (Spain), the Isle of Sylt (Germany), and Bergen (Norway). Clitellate specimens were purchased from commercial providers. A detailed list of the investigated annelid species and respective collection sites is provided in table 1. Cockroaches of the species *Leucophaea maderae *FABRICIUS, 1792 were bred in laboratory cultures. The principal neuroarchitecture of the brain was revealed by a combination of immunohistochemistry and cell nuclei labeling performed on free floating vibratome sections as described in Heuer and Loesel [25] (2008).

**Table 1:** List of investigated annelid species, including collection sites, ecological background information from sampling localities and literature as well as number of investigated specimens.

Species	Habitat, depth, sampling locality	Biology **(from Fauchald and Jumars **[62]**)**	Investigated specimens
*Aphrodita aculeata* LINNAEUS, 1761	Underneath rocks, 5 m, Cala Olivera, Ibiza (2006, 2007)	Slow-moving carnivore, epibenthic	5

*Arenicola marina* (LINNAEUS, 1758)	Infaunal, on tidal flats, Königshafen, Sylt (2008)	Detritus feeder, burrowing	3

*Branchiomma bombyx* (DALYELL, 1853)	On algae-incrusted rocks, 2-10 m, Cala Llenya, Cala Olivera, Ibiza (2007)	Suspension feeder, tube-dwelling	2

*Eunice torquata* QUATREFAGES, 1865	In crevices and adjacent detritus, 1-15 m, Cala Llenya, Cala Olivera, Cala Vedella, Penyal de s'Aguila, Ibiza (2006, 2007)	Predator, free-living	8

*Eupolymnia nebulosa* (MONTAGU, 1818)	Attached to bottom side of rocks, 1-6 m, Cala Llenya, Cala Olivera, Cala Vedella, Ibiza (2006, 2007)	Detritus feeder, tube-dwelling	9

*Harmothoe areolata* (GRUBE, 1860)	Underneath rocks, 1-6 m, Cala Olivera, Penyal de s'Aguila, Ibiza (2006, 2007)	Carnivorous, epibenthic	5

*Hesione pantherina* RISSO, 1826	Underneath rocks, 1-10 m, Cala Llenya, Cala Olivera, Ibiza (2007)	Carnivorous, free-living	2

*Hirudo medicinalis* LINNAEUS, 1758	Purchased at a local pharmacy, Aachen	Hematophageous, free-living	3

*Lepidonotus clava* (MONTAGU, 1808)	On algae-covered rocks, 1-10 m, Cala Olivera, Penyal de s'Aguila, Ibiza (2006, 2007)	Carnivorous, epibenthic	6

*Lumbricus terrestris* LINNAEUS, 1758	Purchased from a local vendor, Aachen	Detritus feeder, burrowing	12

*Lumbrineris *cf. *fragilis* (MÜLLER, 1776)	Infaunal, 90-200 m, Raunefjord, Hjeltefjord, Bergen (2008)	Carnivorous, epibenthic/burrowing	3

*Neoleanira tetragona* (OERSTED, 1845)	in silt, 90-200 m, Raunefjord, Hjeltefjord, Bergen (2008)	Carnivorous, epibenthic	1

*Nephtys hombergii* SAVIGNY, 1818	Infaunal, on tidal flats, Königshafen, Sylt (2008); in silt, 90-200 m, Raunefjord, Hjeltefjord, Bergen (2008)	Carnivorous, motile burrower	2

*Nereis diversicolor* (MÜLLER, 1776)	Infaunal, on tidal flats, Königshafen, Sylt (2008). Additional specimens, kindly provided by J. von Döhren, were collected on the isle of Helgoland (Germany)	Omnivorous, gallery-building	35

*Odontosyllis *cf. *fulgurans* (AUDOUIN & MILNE-EDWARD, 1833)	On algae-incrusted rocks and in silt samples, 90-200 m, Raunefjord, Hjeltefjord, Bergen (2008)	Carnivorous, epibenthic	4

*Ophelia limacina* (RATHKE, 1843)	Infaunal, 3-12 m, Cala Llenya, Ibiza (2006, 2007)	Detritus feeder	4

*Phyllodoce maculata* (LINNAEUS, 1767)	Infaunal, on tidal flats, Königshafen, Sylt 2008	Hunting predator/scavenger, free-living	3

*Pista cristata* (MÜLLER, 1776)	Attached to hard substrates and in silt, 90-200 m, Raunefjord, Hjeltefjord, Bergen (2008)	Detritus feeder, tube-dwelling	2

*Sabella penicillus* LINNAEUS, 1767	Clump of tubes recovered from a silt sample, 90-200 m, Raunefjord, Hjeltefjord, Bergen (2008)	Suspension feeder, tube-dwelling	4

*Scalibregma inflatum* RATHKE, 1843	In silt, 90-200 m, Raunefjord, Hjeltefjord, Bergen (2008)	Detritus feeder	5

*Sthenelais *cf. *limicola* EHLERS, 1864	Infaunal, 3-12 m, Cala Llenya, Ibiza (2007)	Predator, epibenthic	3

*Thelepus cincinnatus* (FABRICIUS, 1780)	Attached to rocks and in silt, 90-200 m, Raunefjord, Hjeltefjord, Bergen (2008)	Detritus feeder, tube-dwelling	1

*Tomopteris helgolandica* (GREEFF, 1879)	Two specimens recovered from a dredge sample, Raunefjord, Hjeltefjord, Bergen (2008)	Predator, pelagic	2

In short, annelid heads - or, for *L. maderae*, dissected brains - were fixed overnight in 4% paraformaldehyde in 0.1 M phosphate buffered saline (PBS) at room temperature. Prior to paraformaldehyde fixation, specimens intended for anti-histamine immunohistochemistry were fixed overnight in 4% carbodiimide (1-Ethyl-3-(3-dimethylaminopropyl)-carbodiimide hydrochloride; Sigma-Aldrich, Steinheim, Germany) in PBS.

For vibratome-sectioning (VT1000S, Leica Microsystems, Wetzlar, Germany), the tissue was rinsed in four changes of PBS and then embedded in a gelatine/albumin medium. After hardening for 16-18 h in 15% formalin in PBS at 8°C, the gelatine/albumin blocks were cut into sections of 80-100 μm thickness. The sections were washed in six changes of PBS with 0.1% Triton X-100 (TX) and subsequently pre-incubated overnight in a blocking solution of 500 μl PBS containing 0.5% TX and 5% normal swine serum (Jackson ImmunoResearch, West Grove, PA). Primary antibodies were added directly to the blocking solution: anti-FMRF-amide (ImmunoStar, Hudson, WI) at a dilution of 1:20000, anti-serotonin (Sigma-Aldrich, Saint Louis, MO) at a dilution of 1:20000 or anti-histamine (Progen Biotechnik, Germany) at a dilution of 1:50000. Specificity controls for the antibodies were performed by the suppliers by liquid-phase preabsorption of the diluted antisera with 100 μg/ml of FMRF-amide, 200 μg/ml serotonin conjugated to bovine serum albumin, or 10-100 μg/ml histamine, respectively. Immunostainings were completely abolished by these pretreatments. After 26 h of incubation at room temperature, sections were washed in six changes of PBS with 0.1% TX. They were then transferred to 500 μl PBS containing 0.5% TX and 1% normal swine serum. Secondary antibodies (Jackson ImmunoResearch, West Grove, PA) at a dilution of 1:2000 were added directly to this solution and incubated for approximately 16 h at room temperature. Afterwards, the secondary antiserum was removed and the sections were incubated with the nuclear marker DAPI (4',6-Diamidino-phenylindole, dilactate; Sigma-Aldrich, Steinheim, Germany) at a dilution of 1:1000 in PBS for 12 min. Subsequently, sections were rinsed again in several changes of PBS containing 0.1% TX and then mounted on chrome alum/gelatine-coated glass slides under glass coverslips using Elvanol (mounting medium for fluorescent stainings after Rodriguez and Deinhard [29]).

Preparations were analysed with a confocal laser-scanning microscope (TCS SP2, Leica Microsystems, Wetzlar, Germany). A helium/neon laser (excitation wavelength 543 nm, detection range 555-700 nm) was used to detect Cy3 fluorescence, DAPI fluorescence was detected with a diode-laser (excitation wavelength 405 nm, detection range 410-550 nm). Autofluorescence of the tissue was visualized with the argon/krypton laser (excitation wavelength 488 nm, detection range 500-535 nm). Confocal images were finally processed using global imaging enhancement procedures (contrast, brightness) and superposition functions of 'Adobe Photoshop CS'.

Three-dimensional surface reconstruction of cerebral neuropils in *Lepidonotus clava *followed the protocol described in Heuer and Loesel [28] (2009). Using the Amira graphics software package (Mercury Computer Systems Inc., Chelmsford, MA) and a graphic tablet (Intuos3 from Wacom, Krefeld, Germany), neuropil models were rendered from a manually labeled data set of 252 consecutive optical sections in a single, well-preserved specimen.

## Results

**Figure 3:**
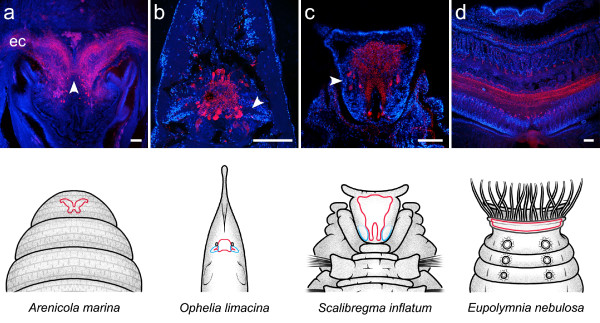
**Neuroanatomy of annelid representatives as revealed by a combination of immunohistochemistry (red) and cell nuclei labeling (blue)**. Schematic drawings depict a dorsal view of the head of the animal, with the brain outlined in red and clusters of small-diameter cells outlined in blue. Immunostainings were produced by the following antisera: anti-serotonin (a, c, d), anti-FMRFamide (b). **Arrowheads **indicate a narrow neuropil band connecting both cerebral hemispheres in (a), lateral protuberances of the central neuropil encased in dense assemblies of minute cell somata in (b), and laterally arranged somata showing serotonin-immunoreactivity in (c). **ec **circumesophageal connective. Scale bars: 100 μm.

**Figure 4:**
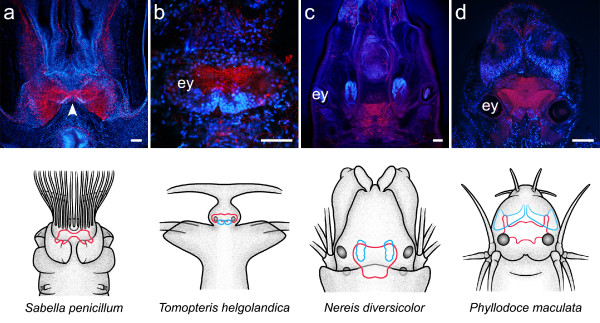
**Neuroanatomy of annelid representatives as revealed by a combination of immunohistochemistry (red) and cell nuclei labeling (blue)**. Schematic drawings depict a dorsal view of the head of the animal, with the brain outlined in red and clusters of small-diameter cells outlined in blue. Immunostainings were produced by the following antisera: anti-FMRFamide (a), anti-serotonin (b, c, d). The **arrowhead **points at the narrow neuropil band connecting both hemispheres of the brain in *S. penicillus*. **ey **eye. Scale bars: 100 μm.

**Figure 5:**
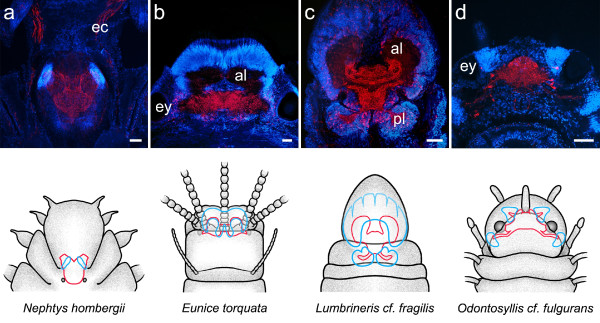
**Neuroanatomy of annelid representatives as revealed by a combination of immunohistochemistry (red) and cell nuclei labeling (blue)**. Schematic drawings depict a dorsal view of the head of the animal, with the brain outlined in red and clusters of small-diameter cells outlined in blue. Immunostainings were produced by the following antisera: anti-serotonin (a, d), anti-FMRFamide (b, c). **al **anterior lobe, **ec **circumesophageal connective, **ey **eye, **pl **posterior lobe. Scale bars: 100 μm.

**Figure 6:**
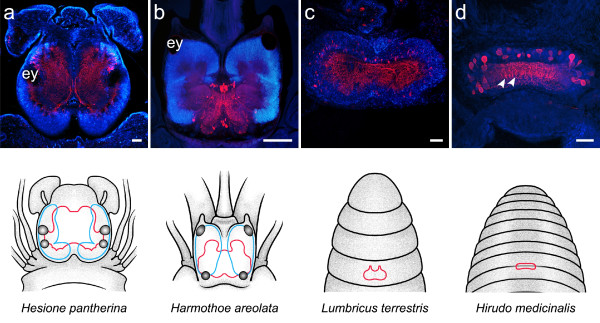
**Neuroanatomy of annelid representatives as revealed by a combination of immunohistochemistry (red) and cell nuclei labeling (blue)**. Schematic drawings depict a dorsal view of the head of the animal, with the brain outlined in red and clusters of small-diameter cells outlined in blue. Immunostainings were produced by the following antisera: anti-FMRFamide (a, b, d), anti-histamin (c). **Arrowheads **point at columnar fiber elements in the anterior part of the central neuropil in *H. medicinalis*. **ey **eye. Scale bars: 100 μm.

**Figure 7:**
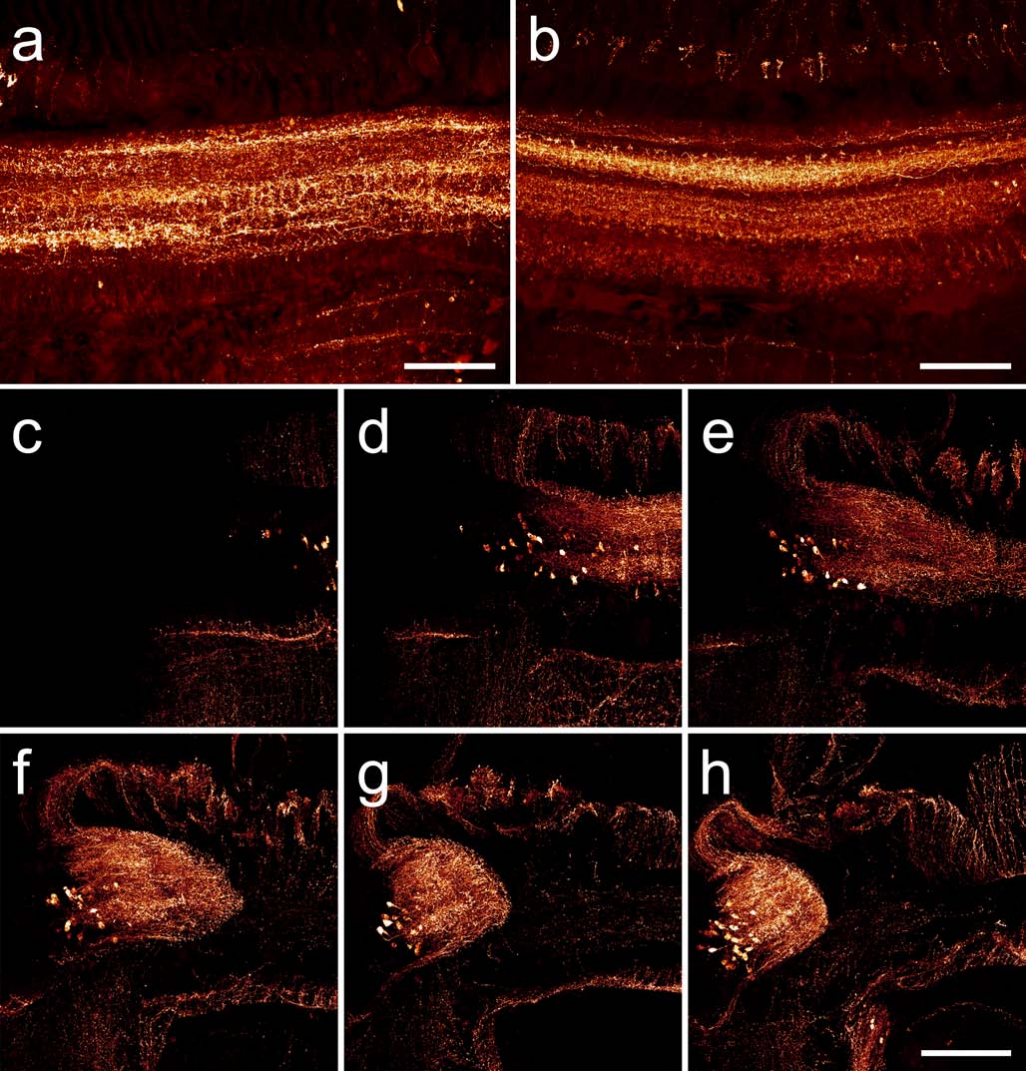
**Neuroarchitecture in terebellid polychaetes**. (a, b) Horizontal sections through the brain of *Eupolymnia nebulosa*. While the ribbon-shaped brain does not contain distinct neuropil compartments, an anterior-posterior stratification is revealed by FMRF-like (a) and histamine (b) immunoreactivity (anterior is towards the top of the picture). (c-h) Consecutive horizontal sections (proceeding ventrad in reading direction) showing serotonin immunoreactivity in the brain of the terebellid polychaete *Thelpus cincinnatus*. Similar to the condition in *E. nebulosa*, the brain in this species is confluent with the circumesophageal connectives, forming a ring-like band around the esophagus. Neuronal somata are located at the outer perimeter of the brain. Scale bars: 200 μm.

**Figure 8:**
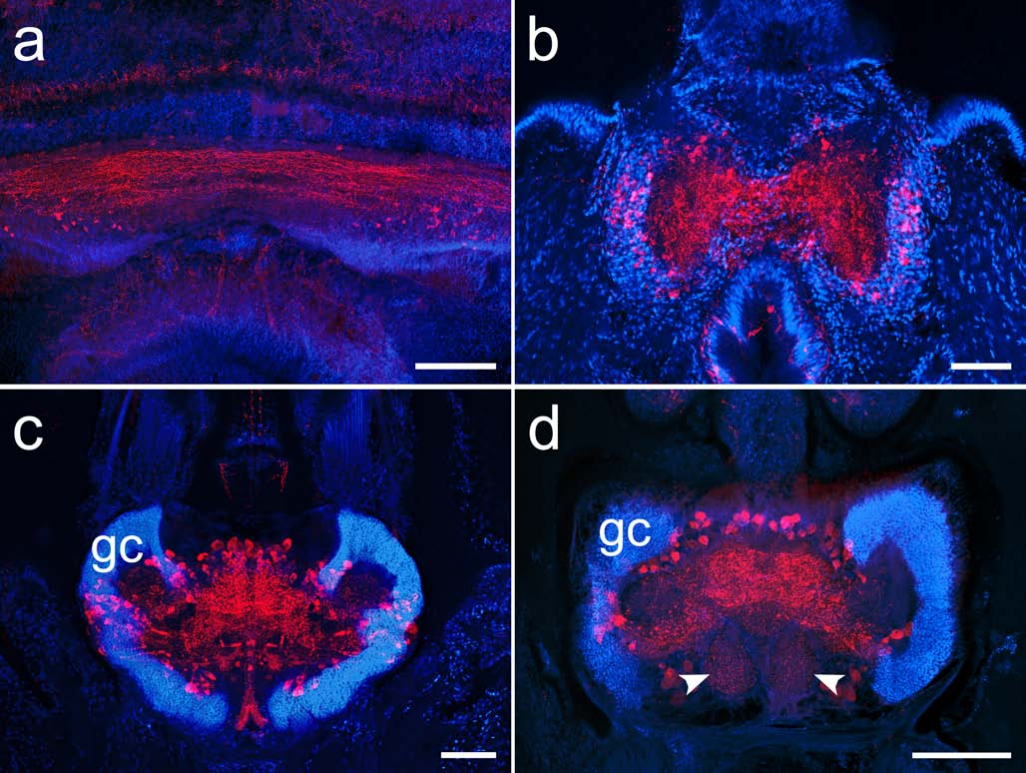
**Neuroanatomy in four different polychaete species**. (a) Horizontal section through the brain of the terebellid polychaete *Pista cristata*. Serotonin immunoreactivity and cell nuclei labelings reveal a close resemblance to the neuroanatomical conditions observed in the terebellid species *E. nebulosa *and *T. cincinnatus*. (b) Neuroanatomy of the brain in the sabellid polychaete *Branchiomma bombyx *as revealed by FMRF-like immunoreactivity. Similar to the condition in *S. penicillus*, the neuropil forms two fiber masses that are dorsally connected by a narrow neuropil band. Homogeneously distributed neuronal somata, some showing FMRF-like immunoreactivity, surround the brain. (c) FMRF-like immunoreactivity in horizontal brain sections of *Sthenelais *cf. *limicola *and (d) *Aphrodita aculeata*. Nuclear markers reveal numerous tightly packed cell bodies (**gc**) that surround the dorsal parts of mushroom body neuropils. **Arrowheads**: clusters of olfactory glomeruli. Scale bars: (a, c, d) 200 μm (b) 80 μm.

**Figure 9:**
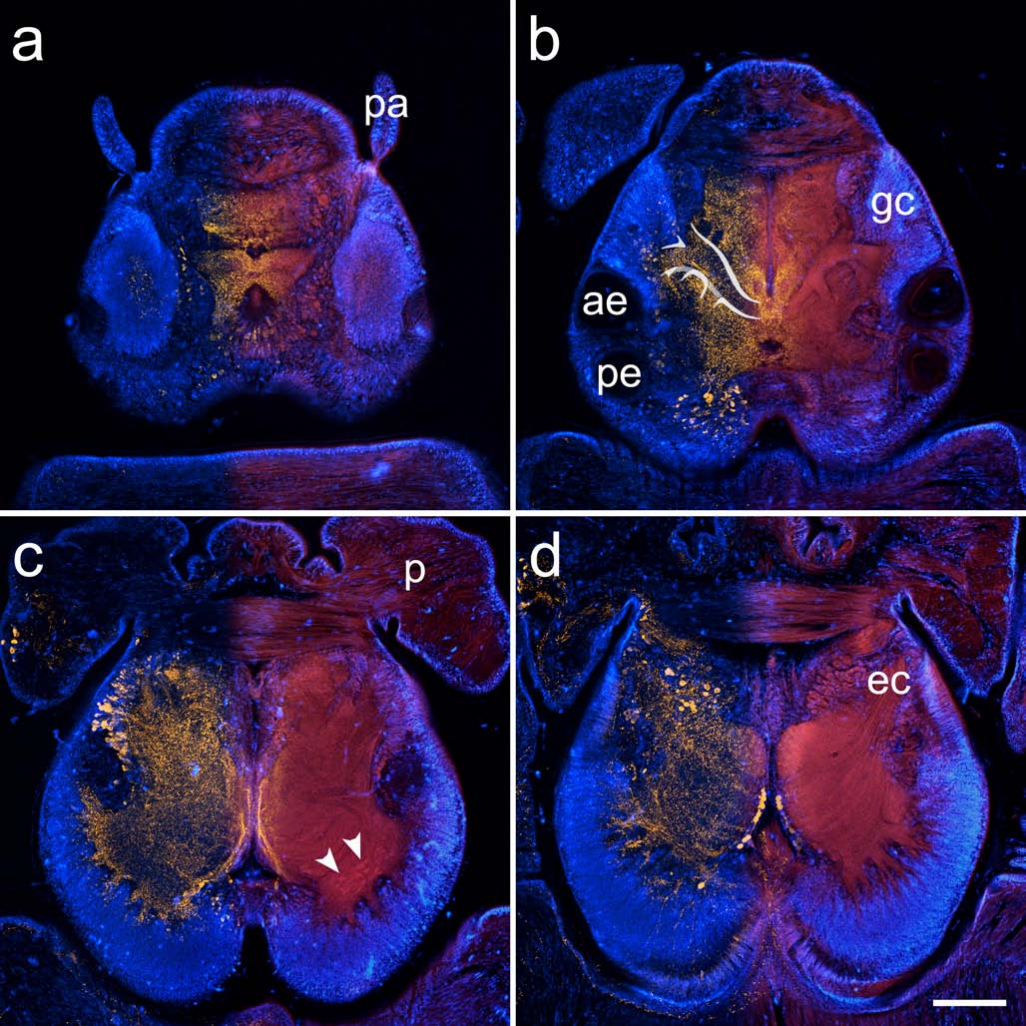
**Consecutive horizontal sections through the head of *Hesione pantherina *(proceeding ventrad from a-d)**. The overlay images show FMRFamide-like immunoreactivity (yellow, left half) and autofluorescence images (red, right half) together with DAPI-labeled cell nuclei (blue). Immunostainings reveal intricate patterns in the dorsal part of the fiber mass (a, b). Paired aggregations of small globuli cells (**gc**) are situated in front of the anterior eyes (**ae**) and give rise to stalk-like neuropils converging at the midline of the brain. While the central parts of these neuropils are nearly devoid of immunostaining, scattered FMRFamide-like immunoreactivity can be observed in the peripheral parts (b). Ventrally, the globuli cell mass extends posteriorly, surrounding the central fiber mass, which is now clearly separated into two hemispheres. Small spheroid subcompartments, reminiscent of olfactory glomeruli, can be discerned in the posterior region of each hemisphere (**arrowheads**). Autofluorescence also reveals a striation of the nervous tissue in the ventral part of the brain. **ec **circumesophageal connective, **p **palps, **pa **prostomial antennae, **pe **posterior eyes. Scale bar: 80 μm.

**Figure 10:**
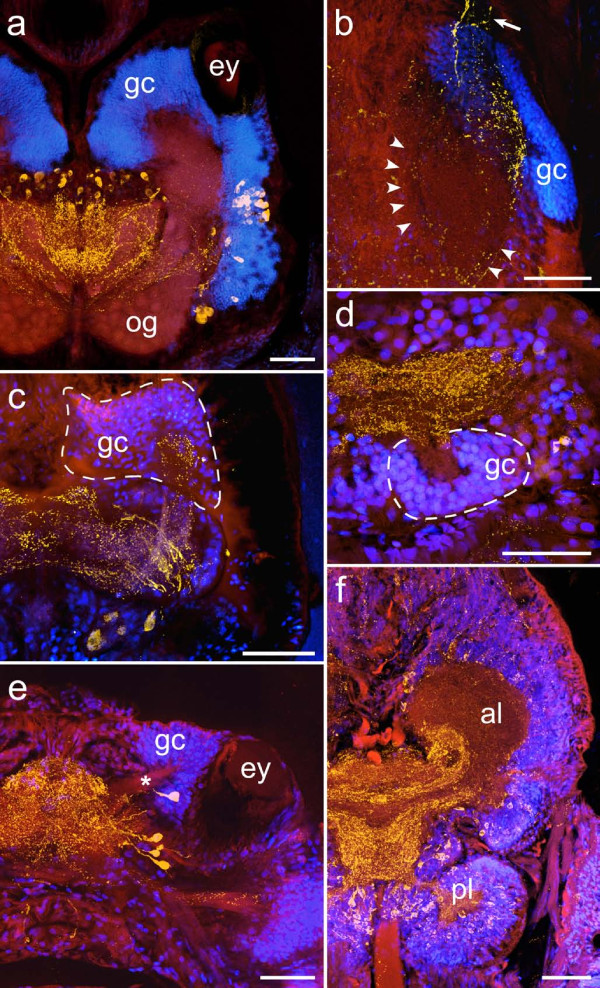
**Overlay images combining autofluorescence (red), immunoreactivity (yellow; antiserum directed against FMRFamide in a, b, f, and against serotonin in c, d, e) and nuclear labeling (blue) to reveal mushroom body neuropils in (a) *Harmothoe areolata *(b) *Nepthys hombergii *(c) *Phyllodoce maculata *(d) *Tomopteris helgolandica *and (e) *Odontosyllis *cf. *fulgurans***. The cell cortex of the brain of *Lumbrineris *cf. *fragilis *(f) is not considered indicative of mushroom body neuropils due to the homogeneous distribution of somata and the lack of a distinctive associated neuropil compartment. **al **anterior lobe, **ey **eye, **gc **globuli cells, **og **olfactory glomeruli, **pl **posterior lobe, **Arrowheads **mark the poorly delineated neuropil boundary in *N. hombergii*, **dashed lines **mark the boundaries of globuli cell aggregations. **Arrow **root of the circumesophageal connective. Scale bars: 80 μm.

**Figure 11:**
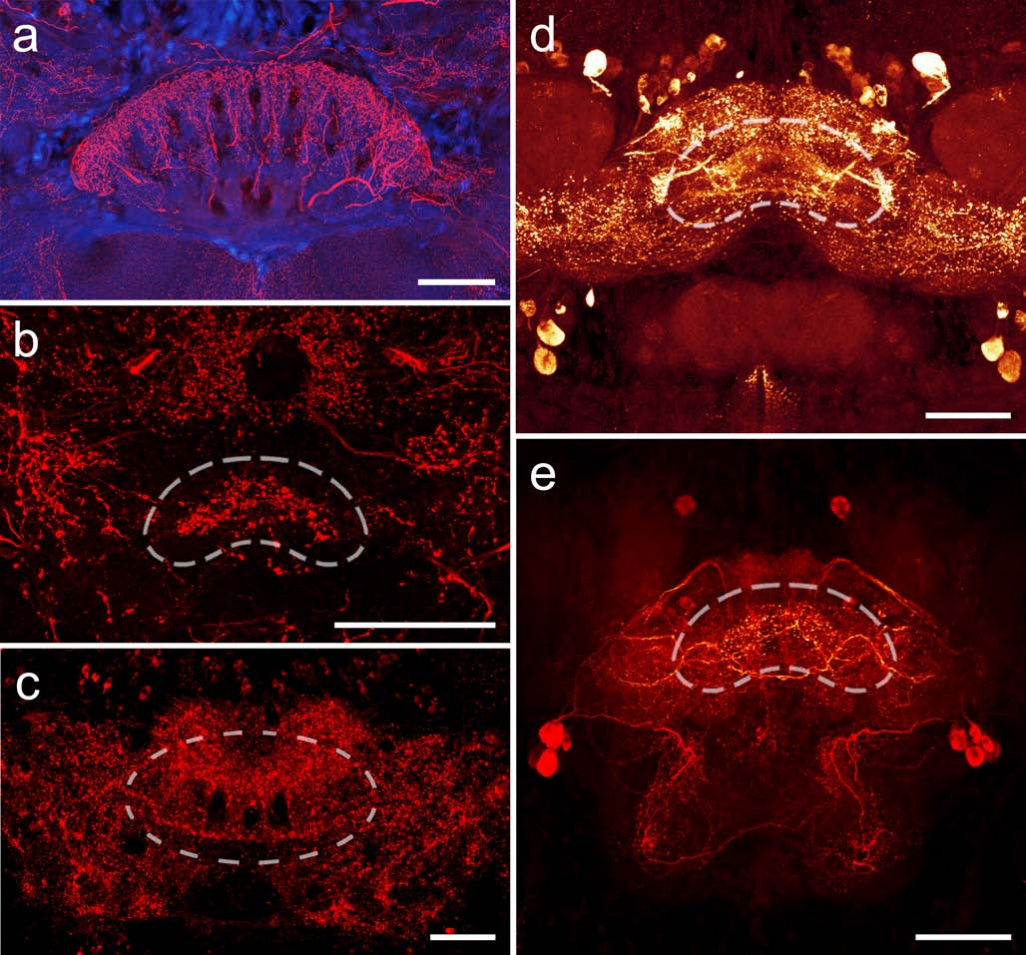
**Comparison of unpaired midline neuropils in an arthropod and various polychaete worms**. (a) Serotonin immunoreactivity in the brain of the hexapod *Leucophaea maderae*. The central body of the arthropod brain is a characteristic unpaired midline neuropil comprising columnar and tangential components. (b) Histamine immunoreactivity reveals a small, crescent-shaped neuropil in the brain of *Nereis diversicolor*. (c) FMRFamide-like immunoreactivity shows a neuropil composed of tangential and columnar elements in the brain of the polychaete *Eunice torquata*. Tangential fibers projecting from laterally arranged cell bodies give rise to crescent-shaped fiber tangles in the brain of *Harmothoe areolata *(d, FMRFamide immunoreactivity) and *Lepidonotus clava *(e, serotonin immunoreactivity). Scale bars: 80 μm.

Immunohistochemical methods were used to analyze brain anatomy and reveal neuropil substructures in representatives of 22 annelid species(see Table 1). Additionally, immunostained sections of an arthropod brain (*Leucophaea maderae*, Insecta) are presented for comparative purposes (Fig. 1). A 3D rendering of neuropils encountered in the polychaete *Lepidonotus clava *is presented in Fig. 2. Figs. 3, 4, 5, and 6 summarize the occurrence and anatomy of distinctive cerebral neuropils across the range of the investigated species. Results are shown in exemplary horizontal sections of immunohistochemical preparations, along with schematic drawings depicting characteristic neuroanatomical features for each of the investigated species. Additional figure plates present neuroanatomical similarities in closely related species (Figs. 7, 8) as well as details on neuropil substructures (Figs. 9, 10, 11).

### *Arenicola marina *(Arenicolidae, Scolecida, Polychaeta)

The brain of the lugworm *A. marina *is formed by the dorsal convergence of the paired circumesophageal connective. It consists of two fiber masses that are clearly separated from each other and are only posteriorly connected by sparse fibers bundles (Fig. 3a). The brain does not contain distinct subcompartments. Neuronal somata reside dorsally and laterally of the fiber masses; conspicuous aggregations of small diameter cells are not apparent.

### *Ophelia limacina *(Opheliidae, Scolecida, Polychaeta)

*O. limacina *is an infaunal substrate-feeding species with a small, cone-shaped prostomium. The brain has an oval to round shape and is situated between two cerebral eyes (Fig. 3b). It does neither contain lobed neuropils nor any other distinct subcompartments. Instead, serotonin- and FMRF-immunoreactivity reveal the brain to consist of an undifferentiated neuropil tangle that is surrounded by medium to large-sized neuronal somata. Additionally, two populations of comparatively small cell bodies are located bilaterally of the posterior part of the neuropil. They form a pair of cone-shaped, laterally tapering aggregations that encase small protuberances of the central neuropil.

### *Scalibregma inflatum *(Scalibregmatidae, Scolecida, Polychaeta)

In the sediment-dwelling species *S. inflatum*, the brain is located in the middle of the T-shaped prostomium (Fig. 3c). It consists of a central neuropil that is dorsally and laterally covered by neuronal somata. While numerous of these somata exhibit FMRF-like immunoreactivity, serotonin immunoreactivity is restricted to a limited number of cell bodies, most of which are located to both sides of the brain at its anterior-posterior midline (Fig. 3c). The central neuropil forms a compact fiber mass anteriorly, which gradually tapers into two medially separated lobes posteriorly. Distinct neuropil substructures are not distinguishable within the fibrous meshwork. Laterally of the posterior lobes, loosely packed aggregations of small cell bodies occupy the dorsal part of the prostomium. The border of these cell clusters is diffuse and they are not invaded by protrusions of the central neuropil, nor do they surround any neuropil components.

### *Eupolymnia nebulosa *(Terebellidae, Canalipalpata, Polychaeta)

The brain of the sedentary polychaete species *E. nebulosa *is situated above the pharynx and has a ribbon-like shape (Fig. 3d). It is confluent with - and hardly distinguishable from - the circumesophageal connectives, thus forming a ring-shaped neuronal mass around the pharynx (a similar condition is observed in *Thelepus cincinnatus*, see Fig. 7c-h). The brain itself consists of a neuropil band, which is dorsally and posteriorly covered by neuronal somata. The neuropil band is formed by a meshwork of tangled neurites. Immunoreactivity towards different antisera reveals a distinct stratification within the neuropil band (Fig. 7a, b), possibly reflecting functional subdivisions of the brain.

Ribbon-shaped brains with a comparatively simple architecture have also been observed in the terebelliform polychaete species *Pista cristata *and *Thelepus cincinnatus *(Fig. 7c-h, Fig. 8a).

### *Sabella penicillus *(Sabellidae, Canalipalpata, Polychaeta)

The feather duster worm *S. penicillus *is a tube-dwelling filter feeder. The brain of this sedentary polychaete is located at the base of the tentacle crown. It is composed of two bilaterally arranged fiber masses that are dorsally connected by a narrow neuropil band (Fig. 4a). The neuropil appears undifferentiated and contains no distinct subcompartments. Neuronal somata cover the brain neuropil laterally and dorsally but do not form conspicuous aggregations.

Similar neuroanatomical conditions are exhibited by the closely related sabellid species *Branchiomma bombyx *(Fig. 8b).

### *Tomopteris helgolandica *(Tomopteridae, Aciculata, Polychaeta)

In the pelagic predator *T. helgolandica*, the brain is situated between a pair of simple lens- eyes at a posterior position in the prostomium (Fig. 4b). The brain has an oval to rectangular shape and consists of an undifferentiated central neuropil which is covered by medium-sized neuronal somata on its dorsal side. At the posterior margin of the brain, two roughly hemispherical aggregations of small-diameter cell somata are discernable. These globuli cell clusters surround small protrusions of the central neuropil that are partly invaded by serotonin-immunoreactive fibers (Fig. 10d). There is no evidence for lobed neuropils associated with the globuli cell aggregations within the central neuropil.

### *Nereis diversicolor *(Nereididae, Aciculata, Polychaeta)

The neuroanatomy of *N. diversicolor *has already been described in detail in an earlier account [25]. The brain has a roughly trapezoid shape and is located between the two pairs of eyes at a posterior position in the prostomium (Fig. 4c). The most prominent structures in the brain of *N. diversicolor *are two aggregations of globuli cell somata that give rise to clearly demarcated mushroom body neuropils (Fig. 1). They are situated at the dorsal, anterior part of the brain and form a larger anterior and a smaller posterior cluster. They surround the apical parts of the mushroom body neuropil, which forms finger-like protrusions within the globuli cell aggregation. Emanating ventrally from the globuli cells, the protrusions merge into a massive stalk, which bends towards the center of the brain to form two terminal lobes. Associated with the mushroom body neuropils are two clusters of poorly distinguishable glomerular neuropils which receive sensory input from the prostomial palps. The second distinctive subcompartment in the brain of *N. diversicolor *is the optic neuropil. Connecting the four eyes of the animal, the dense neuropil resembles an 'H' with its vertical lines bended outwards [compare [25]]. While its middle part is divided into an anterior and a posterior layer, the optic neuropil is otherwise devoid of neuroarchitectural substructures. Adjacent to the optic neuropil, histamine immunoreactivity reveals an additional unpaired midline neuropil consisting of a crescent-shaped, undifferentiated fiber tangle (Fig. 11b). However, evidence for a possible connectivity between this small, crescent-shaped neuropil and the optic neuropil is lacking.

### *Phyllodoce maculata *(Phyllodocidae, Aciculata, Polychaeta)

The brain of the predatory and highly motile paddle-worm *P. maculata *is situated between - and slightly anterior of - the two eyes of the animal (Fig. 4d). The dorsal part of the brain comprises a dense tangle of neurites that derive from peripherally arranged neuronal somata. Cell nuclei labeled sections reveal two aggregations of comparatively small cell bodies anteriorly of the central neuropil. As the cell bodies form only a loose assembly, the borders of the cell clusters appear rather diffuse. Ventrally, two thick stalk-like fiber-bundles emanate from the globuli cell clusters and enter the brain (Fig. 10c). They extend posteriorly and then, at the level of the eyes, bend inwards. In bending towards the center of the brain, the stalks appear to split into two parts. Poorly delineated from the rest of the neuropil, these parts are possibly confluent with their contralateral counterparts.

### *Nephtys hombergii *(Nephtyidae, Aciculata, Polychaeta)

The brain of the free-living, predatory polychaete *N. hombergii *is displaced posteriorly and occupies a position between the prostomium and peristomium. It has a roughly trapezoid shape and is flanked by a pair of cerebral eyes posteriorly (Fig. 5a). The central part of the brain comprises a largely undifferentiated neuropil which gives rise to the circumesophageal connectives anterio-laterally. A pair of poorly delineated neuropil subcompartments is situated dorsally of the roots of the circumesophageal connectives (Fig. 10b). Barely discernable, these two oval-shaped neuropil regions lie adjacent to aggregations of small diameter cell bodies located at the anterio-lateral borders of the neuropil. In contrast to the neuropil regions, the globuli cell clusters are well defined and exhibit a sharp boundary.

### *Eunice torquata *(Eunicidae, Aciculata, Polychaeta)

In the carnivorous species *E. torquata*, the brain is located at an anterior position in the prostomium. The neuropil consists of a main fiber mass that is situated between the eyes of the animal and two anterior lobes lying in front of that fiber mass (Fig. 5b). Immunoreactivity towards FMRF-amide and histamine antisera is largely restricted to the main part of the brain, with the anterior lobes exhibiting only a comparatively weak signal. While neurites in the main fiber mass form a tangled meshwork for the most part, histamine as well as FMRF-like immunoreactivity reveal individual fibers to form an unpaired midline neuropil (Fig. 11c). The midline neuropil is slightly curved posteriorly and comprises distinct columnar components that connect two commissural fiber bundles.

In *E. toquata*, a huge mass of densely assembled, minute cell bodies is situated in front of the brain. Dorsally, these somata form an unpaired aggregation that is divided into an anterior and a postior part. In contrast to the anterior part, many cells of the posterior part exhibit FMRF-like immunoreactivity. Proceeding ventrad, the posterior part gradually diminishes, and the anterior part separates to form bilaterally paired clusters that surround the anterior lobes. Despite the presence of a large aggregation of minute cells, neither the main part of the brain, nor the anterior lobes contain distinct subcompartments.

### *Lumbrineris cf.fragilis *(Lumbrineridae, Aciculata, Polychaeta)

The brain of the infaunal selective deposit feeder *L*. cf. *fragilis *is situated at a posterior position in the cone-shaped prostomium. It is composed of a main neuropil and a set of large anterior and small posterior lobes (Fig. 5c). The cerebral neuropil is almost completely enveloped by a cortex of small-diameter cell bodies. The densely packed somata form confluent aggregations that surround the anterior lobes, the main neuropil, and the posterior lobes on all sides (Fig. 10f). They do, however, not give rise to any distinctive neuropil substructures. Individual cell bodies in the surrounding cell cortex show immunoreactivity withantisera directed against FMRF -amide and serotonin. Immunopositive cell bodies were observed to be associated with all three parts of the brain, but were most numerous in the posterior lobes. Immunoreactivity within the neuropil was most pronounced in the central part of the brain, where commissural fiber tracts and large parts of the neuropil were stained (Fig. 10f). Strong immunostaining could also be observed in the posterior lobes. In contrast, antisera produced only faint and scattered immunostainings in the anterior lobes.

### *Odontosyllis cf. fulgurans *(Syllidae, Aciculata, Polychaeta)

The brain of *O*. cf. *fulgurans*, a predator with a bioluminescent epitokous form, is situated in the anterior part of the prostomium and is located between the eyes of the animal (Fig. 5d). It comprises a triangular to diamond-shaped fiber mass that appears largely undifferentiated and contains no discernable subcompartments, as well as peripherally arranged neuronal somata. Cell nuclei labeled sections show two aggregations of minute cells in each hemisphere. They form a wedge-shaped anterior cluster between the eyes and the anteriorwall of the prostomium, and a posterior cluster situated laterally behind the eyes (Fig. 10e). The anterior clusters encase small, branching protuberances of the neuropil. In a similar fashion, the neuropil also sends out extensions towards the posterior clusters.

### *Hesione pantherina *(Hesionidae, Aciculata, Polychaeta)

The brain of the carnivorous *H. pantherina *is situated roughly in the middle of the prostomium and is flanked by two pairs of eyes posteriorly (Fig. 6a, Fig. 9a-d). The cerebral fiber massis well differentiated. Its dorsal part contains two elaborate neuropils that are associated with paired aggregations of minute cell bodies situated in front of the anterior eyes (Fig. 9a, b). The neuropils have a stem-like shape, formed by the fusion of several lateral branches that converge towards the midline of the brain. The lateral branches of the neuropil exhibit FMRF-like immunoreactivity. FMRF-amide antiserum also produces characteristic patterns in the rest of the dorsal fiber mass. Ventrally, the cerebral fiber mass is divided into almost completely separated hemispheres (Fig. 9c, d). The hemispheres are laterally and posteriorly surrounded by densely assembled cell bodies. These aggregations appear to be confluent with the dorsal globuli cell clusters and give rise to distinct, converging fiber bundles that merge with the main fiber mass. Small clusters of poorly demarcated spheroidal compartments are barely distinguishable in the posterior region of the fiber mass (Fig. 9c). The ventral part of the fiber mass also shows a slight striation (Fig. 9d).

### *Harmothoe areolata *(Polynoidae, Aciculata, Polychaeta)

The brain of the predatory scaleworm *H. areolata *is dominated by a pair of huge mushroom bodies (Fig. 6b). A detailed morphological description of these neuropils has already been provided elsewhere [see [28]]. Emerging from two large aggregations of small diameter globuli cells located dorsally in the prostomium, each mushroom body neuropil forms a well-defined lobe anteriorly and several median extensions posteriorly. The latter establish a close connection not only with the central neuropil but also with a clearly delineated cluster of glomeruli lying in the ventro-posterior part of the brain. A second group of glomeruli is situated within the anterior lobe of the mushroom bodies where it most likely receives input from the palpal nerve. The central neuropil itself consists of a largely undifferentiated meshwork of fibers. However, FMRF-amide-like immunoreactivity reveals a small, crescent-shaped neuropil concealed within the meshwork. This unpaired, poorly delineated neuropil is formed by several neurites that converge at the brain's midline.

Large and well-defined mushroom bodies showing a similar organization have also been observed in the polynoid representatives *Lepidonotus clava *(Fig. 2) and *Sthenelais *cf. *limicola*as well as in the aphroditid species *Aphrodita aculeata *and *Neoleanira tetragona*. Further neuroarchitectural similarities among scaleworm species include the presence of glomerular clusters (Fig. 2, Fig. 10a) and aggregations of fibers forming unpaired midline neuropils (Fig. 2, Fig. 11d, e).

### *Lumbricus terrestris *(Lumbricidae, Oligochaeta, Clitellata)

The brain of the substrate feeding earthworm *L. terrestris *is dislocated posteriorly and lies on top of the esophagus in the third body segment. It is bilobed in shape, with each of the hemispheres sending out a prostomial nerve anteriorly (Fig. 6c). The brain is composed of a central neuropil and a dense cortex of neuronal somata that are not forming conspicuous clusters. The central fiber mass does not contain discernable neuropil subcompartments.

### *Hirudo medicinalis *(Hirudinidae, Hirudinea, Clitellata)

The brain of the parasitic leech *H. medicinalis*is situated posteriorly to the medi an jaw in the fifth body segment (Fig. 6d). The brain is comparatively small and of a ribbon-like shape. Its central fiber mass is weakly differentiated into an anterior stratum comprising mostly columnar fiber elements and a posterior stratum comprising transverse fiber bundles. Neuronal cell somata are comparatively large and are arranged homogeneously around the cerebral neuropil. Dense aggregations of small-diameter neuronal somata are lacking and the central fiber mass is devoid of discernable substructures.

### *Leucophaea maderae *(Blaberidae, Dictyoptera, Insecta)

The neuroanatomy of the cockroach *L. maderae *largely corresponds to the ground pattern found in pterygote insects [for an overview see e.g. [30,31]]. Distinctive neuropils of the brain are the paired mushroom bodies (Fig. 1a) and the unpaired central body (Fig. 9a). Immunoreactivity towards a Horseradish-peroxidase antiserum reveals the principal organization of the mushroom body neuropils: Aggregations of tightly assembled globuli cell somata(i n insects termed Kenyon cells) give rise to a neuropil that shows a characteristic subdivision into a dorsal calyx region, embedded within the globuli cell bodies, a stalk-like peduncle, and an arrangement of terminal lobes.

The central body is an unpaired neuropil that spans the midline of the brain. It is part of the central complex, an assembly of interconnected neuropils situated roughly at the center of the brain. Within the central body, neurites are arranged in distinctive columnar and tangential fiber bundles.

## Discussion

The current study presents data obtained from immunohistochemically stained preparations of representatives of more than 20 annelid taxa. Antisera against the near-ubiquitous neuroactive substances serotonin, FMRF-amide, and histamine were used in combination with a nuclear marker to label limited subsets of neurons in the brain. Immunohistochemistry did not aim at identifying the transmitter composition of the brain but at revealing the presence and architecture of higher cerebral centers.

The most prominent neuropil structures to be encountered in the annelid brain are the paired mushroom bodies that occur in a number of polychaete representatives. Mushroom bodies could in some cases be demonstrated to be closely associated with clusters of spheroid neuropils reminiscent of arthropod olfactory glomeruli. Less distinctive subcompartments of the annelid brain are unpaired midline neuropils which have been encountered in several polychaete representatives and which bear a remote resemblance to similar components in the arthropod brain.

### Mushroom bodies

Mushroom bodies are lobed neuropils that are formed by the processes of thousands of small-diameter globuli cells located dorsally in the invertebrate central nervous system [32]. In arthropods, mushroom bodies are usually easily recognizable not only due to their characteristic shape [33], but also due to the fact that glial sheaths clearly delineate the neuropil from the surrounding neuronal tissue [34]. Incontrast to insects, the absence of cell nuclei dispersed along the borders of polychaete mushroom body neuropils (Figs. 1, 9, 10) indicates a lack of likewise well-developed glial boundaries in the brain. Thus, borders of cerebral subcompartments like the mushroom bodies are often only barely perceptible by differences in tissue density and structure (Figs. 9, 10). Among the investigated species, clearly demarcated mushroom bodies have been observed in *Nereis diversicolor *and the scale worm species *Harmothoe areolata*, *Lepidonotus clava*, *Sthenelais *cf. *limicola*, and *Aphrodita aculeata*. In these species, densely assembled globuli cells form well-defined aggregations in the dorsal part of the brain, which give rise to lobed neuropils that are clearly distinguishable within the cerebral fiber mass (Fig. 8c, d, Fig. 10a).

Elaborate mushroom bodies are also present in the brain of *Hesione pantherina*, but show a slightly different organization. They also originate from dorsal aggregations of globuli cells, but instead of forming an arrangement of terminal lobes, the stem-like neuropils of both hemispheres appear to be contiguous, forming a continuum across the midline of the brain (Fig. 8b). This condition bears resemblance to the contiguity of mushroom bodies that is observed in chelicerates and onychophorans [35].

Mushroom body neuropils are less conspicuous in other polychaete species. The dorsal part of the brain of *Nephtys hombergii *contains well-defined globuli cell clusters, but the associated neuropils are only weakly demarcated and barely discernable from the surrounding fiber mass (Fig. 10b). The borders of the globuli cell clusters in *Phyllodoce maculata *appear somewhat diffuse due to the loose assembly of the cell bodies. The boundaries of the stalk-like neuropils that emanate from the globuli cell clusters are also just faintly delineated and become obliterated as the neuropils of both sides converge towards the midline of the brain (Fig. 10c), so that a contiguous mushroom body organization in this species could not be ascertained.

In other polychaetes, lobed neuropil subcompartments are not discernable at all, although conspicuous clusters of small-diameter cells - usually indicative of mushroom bodies - are present. *Odontosyllis *cf. *fulgurans*, *Tomopteris helgolandica*, and the scolecid species *Ophelia limacina *all exhibit clusters of minute cell bodies but lack distinctive neuropils associated with them. Instead, the cell somata assemblies surround protuberances of the cerebral neuropil that extend into the core of the clusters. The protuberances can be small and undifferentiated, as observed in *T. helgolandica *(Fig. 10d) and *O. limacina *(Fig. 3b), or can show minor branching patterns, as evident in the anterior cluster of *O*. cf. *fulgurans*(Fig. 10e). The occurrence of two completely separated cell clusters, situated anteriorly and posteriorly in each hemisphere, is an exceptional characteristic of the brain of *O*. cf. *fulgurans*. *T. helgolandica *and *O. limacina *exhibit only a single cell clusters per hemisphere, situated at a posterior position in the brain. The posterior location of these presumptive globuli cell clusters, as well as the apparent lack of associated neuropil subcompartments within the main fiber mass of the brain must be addressed as a possible pitfall in the identification of mushroom bodies in these species. With respect to the lack of a distinct neuropil, the observed structures might be interpreted as primordial, poorly differentiated mushroom bodies, similar to the mushroom body-like structures that have been described in polyclad platyhelminthes [32,36-38]. However, contrary to the condition in *T. helgolandica *and *O. limacina*, the globuli cells in platyhelminthes are reported to reside at the anterior-lateral border of the brain. Given the lack of a general, specific marker for globuli cells [39], tests for a possible immunoreactivity of the globuli-like cells against taurine, aspartate, and glutamate might provide a means to clarify the true identity of these neural structures in the polychaetes. In insects, antibodies directed against these neuroactive amino acids have been shown to produce immunostaining in specific subpopulations of globuli cells [40]. However, preliminary studies employing taurine-antisera on vibratome sections of *N. diversicolor *generate d only diffuse and probably unspecific staining in the mushroom bodies (unpublished observation). Thus, evidence for the presence of mushroom bodies in *O*. cf. *fulgurans*. *T. helgolandica *and *O. limacina *remains inconclusive so far.

A specific marker for globuli cells would also be beneficial for a closer investigation of the dense cell body assemblies that have been observed in two more polychaete taxa, *Eunice torquata *and *Lumbrineris *cf. *fragilis *(Fig 5b, c, Fig. 10f). In these species, minute cells show a significantly different distribution than in other polychaete representatives. The cell aggregations are not restricted to comparatively small regions of the brain but form a single mass that surrounds the anterior part of the cerebral fiber mass in *E. torquata*, and in *L*. cf. *fragilis *extends to encase the fiber mass almost completely. Due to their predominant distribution and the absence of distinctive neuropils within the brain, the cell clusters cannot be regarded as indicative of the presence of mushroom body-like neuropils in these species at the present state.

Neuroanatomical analyses provided no evidence for the occurrence of mushroom body neuropils in *Arenicola marina*, the terebellid species *Eupolymnia nebulosa*, *Pista cristata*, and *Telephus cincinnatus*, the sabellid species *Branchiomma bombyx *and *Sabella penicillus*, as well as in the clitellate representatives *Hirudo medicinalis *and *Lumbricus terrestris*.

Detailed investigations in *N. diversicolor*, *H. areolata*, and *L. clava *have shown that mushroom bodies in these species share many neuroarchitectural similarities with their arthropod namesakes [25,28]. Recently, such anatomy-derived homology assessments have been backed up by developmental studies investigating gene expression patterns in larvae of the nereid polychaete *Platynereis dumerilii *[27]. The brain of adult *P. dumerilii *contains mushroom body neuropils that are strikingly similar to those of *N. diversicolor*. During ontogeny, the mushroom body anlagen of *P. dumerilii *are observed to express the same combination of genes that is characteristic for the developing mushroom bodies of *Drosophila melanogaster*, namely *dachshund *(*Dach*), *pax6*, *brain factor 1 *(*BF1*), and *seven up *(*Svp*) in the absence of *eyes absent *(*Eya*) and *sine oculis *(*So)*. The investigation of additional genes reported to be expressed in the mushroom bodies of *D. melanogaster *reveals further similarities in the 'molecular fingerprint' of the mushroom bodies. In light of the 'new animal phylogeny' [41], these studies provide independent and therefore significant support for the homology of globuli cells that constitute mushroom bodies in annelids and arthropods. If true, and mushroom bodies in widely separated phyla are indeed homologous and not the product of a purely convergent evolution [see [42]], they probably constitute ancient features of the bilaterian brain.

### Olfactory glomeruli

Olfactory glomeruli are spheroidal neuropils that represent first order integration centres for odour information [43]. Usually occurring in clusters, such neuropils have been described in arthropods, molluscs, and vertebrates [44]. The occurrence of olfactory glomeruli has also been reported for the polychaete species *Arctonoe vittata *GRUBE, 1855 and *Nereis vexillosa *GRUBE, 1851, where they relay information to mushroom body neuropils [32]. The observations of the current study are largely in accordance with these descriptions, as olfactory glomeruli could be identified in nereid *Nereis diversicolor *and the scale worm species *Harmothoe areolata*, *Lepidonotus clava*, and *Aphrodita aculeata*. Furthermore, small globular subcompartments possibly representing olfactory glomeruli were also discovered in the brain of *Hesione pantherina *(Fig. 9c). Considering that in arthropods, olfactory glomeruli provide the predominant sensory input to the mushroom bodies [32], it is hardly surprising that the occurrence of olfactory glomeruli in the polychaete brain coincides with the presence of well-developed mushroom bodies. However, just like the mushroom body neuropils, olfactory glomeruli were observed to show varying degrees of differentiation between different species. The most pronounced glomerular clusters were observed in *H. areolata*, while the glomeruli in *L. clava *and *A. aculeata *were less clearly demarcated. In *N. diversicolor*, the presence of olfactory glomeruli could only be confirmed by retrograde tracing experiments (unpublished observation). Thus, the occurrence of poorly differentiated olfactory glomeruli in the brain of polychaetes that possess equally poorly differentiated mushroom bodies (e.g. *Nephtys hombergii*) cannot be ruled out. Moreover, the possibility of a direct innervation of the mushroom bodies, as demonstrated for *N. diversicolor *[25], provides an alternative pathway for the transmission of odor information to the mushroom body neuropils that might also be utilized in other polychaete species.

The occurrence of two separate clusters of olfactory glomeruli in each brain hemisphere of *H. areolata *[compare [28]] is an exceptional neuroarchitectural condition not observed in any other of the investigated species. The discovery of olfactory glomeruli recieveing terminals of the palpal nerve and residing in close proximity to the mushroom body peduncle in *N. diversicolor *(unpublished observation) are reminiscent of the arrangement of the anterior glomerular cluster in *H. areolata*. This gives rise to speculations about the sensory input to the second, posteriorly located glomerular cluster in *H. areolata*. It seems conceivable that the posterior glomeruli are also involved in the processing of olfactory cues, possibly receiving chemosensory input from the nuchal organs situated at the posterior margin of the prostomium [45] and relaying information to the mushroom bodies. However, our immunostainings did not provide data to confirm such a pathway.

### Unpaired midline neuropils

In this account, the general term 'unpaired midline neuropil' is applied to hitherto unspecified neuropils that span the midsagittal plane of the brain; the term does not imply homology between individual unpaired midline neuropils encountered in different species.

Unpaired midline neuropils have been observed in only a limited number of the investigated annelid species (Fig. 11). This might well be due to the fact that these cerebral substructures - in contrast to arthropod midline neuropils, such as the tetraconate central body or the chelicerate arcuate body - do not show definite boundaries in the form of glial sheaths. Instead, the midline neuropils were found to be tightly interwoven with the surrounding fiber mass and were only distinguishable if the neuropil showed pronounced immunoreactivity towards the applied antiserum. Unpaired midline neuropils occurred only in polychaete representatives and exhibited different degrees of differentiation. The simplest organization was encountered in *Nereis diversicolor*, where the unpaired midline neuropil consists of a small, crescent-shaped fiber tangle with no apparent internal differentiation (Fig. 11b). A more complex organization was observed in the unpaired midline neuropil of *Eunice torquata*, which comprises commissural elements that are linked by an arrangement of discrete fiber bundles (Fig. 11c). The unpaired midline neuropils in the polynoid species *Harmothoe areolata *and *Lepidonotus clava *are situated in the ventral part of the brain and can be considered homologous due to their architectural commonalities. The neuropils consist of fiber tangles that comprise ramifications of neurites that extend along the lateral axis of the brain, forming characteristic intersections (Fig. 11d, e). Their neuronal somata are located in two groups on both sides of the brain. In *L. clava*, additional fibers could be observed to enter the neuropil perpendicularly (Fig. 11e).

Among the unpaired midline neuropils of the polychaete brain, those observed in *L. clava *and *E. torquata *bear faint resemblance to midline neuropils of the arthropod brain. The perpendicular arrangement of neurite bundles, particularly in *E. torquata*, is reminiscent of the tangential and columnar organization of the central body in tetraconates (Fig. 11a) or the arcuate body in chelicerates. However, in the polychaetes, the fiber bundles do not appear to form distinct layers or the chiasmic patterns that are characteristic for unpaired components in the arthropod central nervous system [35]. Furthermore, the specific connectivities of these neuropils to other parts of the polychaete brain still remain inconclusive, impeding attempts at homologizing these structures with unpaired midline neuropils in arthropods.

### Evolution of annelid brain complexity

For lack of a well-resolved and robust annelid phylogeny, genera were grouped according to the cladistic analysis provided by Rouse and Fauchald [10]. Although recent molecular studies have demonstrated that the major clades proposed by Rouse and Fauchald cannot be regarded as monophyletic groupings [4,14], the provisional classification of the investigated species into Clitellata, Scolecida, Canalipalpata, and Aciculata serves to show that the occurrence of mushroom bodies is largely restricted to aciculate representatives. Outside Aciculata, conspicuous cell clusters possibly indicating the presence of mushroom body neuropils were only observed in the scolecid genus *Ophelia*. However, the similar cell clusters in the closely related *Scalibregma *species were not interpreted as mushroom bodies due to the lack of a core neuropil, discouraging the notion that the structures in *Ophelia *species indeed represent mushroom body neuropils.

Aciculata retains the highest support among the clades proposed by Rouse and Fauchald [4,10,14], containing most of the species that had formerly been united in the grouping 'Errantia'. The presence of mushroom bodies in errant polychaetes - characterized by a motile, often predatory or scavenging lifestyle and equipped with a variety of well-developed sensory organs - seems understandable in light of the presumptive function of these brain centers. Among arthropods, mushroom bodies are best investigated in insects, where they are generally assumed to act as integrative brain centers that play a cardinal role in olfactory processing and spatial, associative, and context-dependent learning and memory [42,46]. Commonalities in the neuroarchitectural organization and integration of annelid and arthropod mushroom bodies point towards a similar function of these neuropils in the brain of aciculate polychaetes. It is easily imaginable how such cognitive abilities could translate into an evolutionary advantage in free-living animals that have to adapt their behavior to changing environmental demands. For example, learning, memory formation, and decision-making have been shown to correlate positively with fitness by increasing foraging success [47] and growth rate [48] in insects. In terms of mushroom body morphology, comparative neuroanatomical studies in beetles have revealed neuropil structure to be linked with the evolution of different feeding ecologies, as generalist feeders usually display larger and more complex mushroom bodies than specialist feeders [33]. In other insects, mushroom body size has been demonstrated to correlate positively with learning abilities and to be influenced by experience [49]. These studies from arthropods indicate that mushroom bodies could be of a similar adaptive value in coordinating prey finding, predator avoidance, mate choice, and other behaviors in errant polychaetes. However, cognitive abilities come at a price, since information processing and storing, as well as the development and maintenance of neural structures involved in learning and memory, are energetically costly [50-52]. The evolutionary tradeoff between information processing capacity and other fitness-related traits [51,52] might provide an explanation for the relatively simple neuroanatomy and the lack of mushroom bodies observed in other polychaetes, as well as in clitellate representatives. In taxa living in comparatively consistent environments, the fitness gain associated with learning and memory abilities can be expected to become negligible in relation to the costs involved in building and maintaining a suitably complex nervous system. This notion finds further support in the apparent dispensability of elaborate sensory structures (e.g. eyes, palps, antennae, cirri, nuchal organs) in most sedentary and infaunal annelid species. Thus, the relatively simple neuroarchitecture observed in infaunal/sedentary annelids with a detritus/suspension feeding ecology can probably be attributed to selective pressures that do not favor the evolution of complex brains.

However, the varying grades of cerebral complexity observed in different annelids give rise to the question whether the simple neuroarchitecture and the lack of mushroom bodies in non-aciculate annelids is to be interpreted as an ancient evolutionary trait, or rather as a result of a secondary reduction in complexity? In keeping with the traditional view of animal evolution, as a gradual progress from simple to ever more complex forms, annelid mushroom bodies could intuitively be interpreted as a derived, unifying feature of aciculate polychaetes. However, one of the central implications of the 'new animal phylogeny' is that metazoan evolution can no longer be regarded as a gradual succession of increasingly complex forms and that secondary simplifications and reversals are probably far more wide-spread than formerly thought.

Regressive changes in neural complexity are commonly attributed to a narrowing of the ecological niche in a monotonous and extremely simplified environment and a cessation of mobile life [53], and can also be observed in sessile, semi-sessile, and parasitic forms of other taxa [54]. The nervous system in none-parasitic cirripedes, for example, is much simpler than that of free-swimming Crustacea, lacking a distinctive tripartition and comprising only an estimated 200 neurons in *Balanus nubilus*[55]. However, the presence of a more elaborate central nervous system in the barnacle cypris larva shows that the simple nervous system of the adult form results from a partly neural degeneration after settlement, and illustrates the need for coordinating a response to settlement cues in the vagile form [56,57]. Similarly, the nervous system of the highly derived, parasitic rhizocephalic cirripeds is largely reduced. Comparable trends can also be observed in bivalve molluscs, in brachiopods, and in parasitic cestodes, illustrating that comparatively simple neuroarchitectures do not necessarily reflect ancestral conditions.

The problem of dealing with secondary character losses is also reflected in the cladistic analysis of Rouse and Fauchald [10], which placed Clitellata at a basal position in the annelid radiation - an inference that was brought about by the extensive lack of morphological structures in clitellates (e.g. palps, nuchal organs, parapodia, etc.) and the indiscriminative 'absent/present' coding in the character matrix [11,13]. In contrast, recent molecular studies [4,14] suggest that clitellates are derived polychaetes, which would render the Polychaeta paraphyletic. However, these studies have also not been able to provide a robust and convincing resolution for the root of the annelid radiation, proposing a basal position of chaetopterids, magelonids, and/or oweniids. A different approach in the quest to identify the most basal annelid taxa lies in outlining evolutionary scenarios to characterize the ground pattern of annelids on the basis of morpho-functional considerations [13,58,59]. Such studies propose that the annelid stem species was a marine organism [13] with a homonomously segmented body, biramous parapodia, pygidial cirri, and a large prostomium with palps and antennae [59]. Among recent annelids, this organization largely corresponds to the bauplan of errant polychaetes.

It thus appears reasonable to attribute the simple neuroarchitecture and the prevalent lack of mushroom bodies in non-aciculate annelids to widespread secondary reductions in cerebral complexity, and to regard aciculate neuroanatomy as referring to the ancestral annelid condition. The presence of mushroom bodies is therefore probably an ancestral trait in annelids, which has been retained in aciculate polychaetes but has been lost in most other annelid taxa - a notion that is also in agreement with the argument presented in the previous paragraph. In this light, the tightly assembled cell somata observed in *Ophelia *might represent vestiges of largely reduced mushroom bodies. Though no neuroanatomical evidence for mushroom bodies could be detected in the investigated sedentary species, reports of mushroom bodies in *Serpula *[21] and in another sabellid (Strausfeld, personal communication) indicate that mushroom bodies have also been retained in some sedentary polychaete species.

Within aciculate polychaetes, evolutionary trajectories of mushroom body neuropils remain enigmatic. Varying grades of mushroom body differentiation in different species have led early authors to propose that polychaete mushroom bodies evolved from simple to increasingly complex structures, culminating in the elaborate neuropils of polynoid polychaetes [21,22]. In the current study, polynoid polychaetes showed indeed the most complex neuroanatomy of all species investigated. However, in light of the arguments presented above, caution seems warranted in inferring phylogenetic implications from this observation. The largest and most elaborate mushroom bodies to be found in arthropods occur in the horseshoe crab *Limulus polyphemus *[60,61] - commonly regarded as a living fossil.

## Competing interests

The authors declare that they have no competing interests.

## Authors' contributions

CMH and RL conceived this study. CMH took the lead in the immunohistochemical stainings, morphological analysis, and writing. CHGM and CT organized collection trips to Ibiza (Spain) and Bergen (Norway), respectively, and aided in collecting and determining polychaete specimens. CMH, CHGM, CT, and RL all contributed to writing the manuscript. All authors read and approved the final manuscript.
